# RAC2 orchestrates immune cell actin remodeling and migration

**DOI:** 10.3389/fimmu.2026.1917716

**Published:** 2026-08-20

**Authors:** Kayleigh A. Berthiaume Fox, Emily R. Galvin, Geoffrey C. Gurtner, Kellen Chen

**Affiliations:** 1 MD-PhD Medical Scientist Training Program (MSTP), University of Arizona, Tucson, AZ, United States; 2 Department of Biomedical Engineering, University of Arizona, Tucson, AZ, United States; 3 Department of Surgery, University of Arizona College of Medicine, Tucson, AZ, United States

**Keywords:** actin polymerization, chemotaxis, fibrosis, inborn errors of immunity (IEI), neutrophil functions, RAC2 GTPase, superoxide production, wound healing

## Abstract

In this review, we comprehensively summarize how RAC2+ innate immune cells contribute to a spectrum of immune dysregulation and disease states across multiple organ systems in humans. Our lab has recently identified *RAC2’s* critical role in immune cell biology in the context of human and murine wound healing and fibrosis, induced by aberrant mechanical signaling. Thus, we sought to investigate the role of RAC2 in human immune cells. Here, we summarize the effects of gain-of-function (GOF), loss-of-function (LOF), and null *RAC2* mutations on neutrophil phenotypes. As such, we propose a novel mechanism for RAC2-dependent biphasic neutrophil migration, critical for these cells in migrating to sites of injury across many disease states, including wound healing and fibrosis. Innate immune cells circulate and home to injury sites during the initial healing phases in all organs, therefore, understanding RAC2 regulation of neutrophil functions may reveal new avenues for systemic immunomodulatory therapies to treat immune dysregulation, and to better characterize mechanisms of wound healing and fibrosis.

## Introduction

Mechanotransduction networks mediate conversion of mechanical cues into intracellular molecular changes, including the integrin-focal adhesion kinase (FAK) pathway, mitogen-associated protein kinases (MAPKs), and Rac/Rho-GTPases (1–5). Our group has shown that mechanical signaling plays a crucial role in skin and pulmonary fibrosis, and that mechanical forces act through the integrin-FAK pathway to mediate fibrotic wound healing and mesenchymal cell functions (3, 6–12). While most mechanotransduction research has focused on tissue-resident cells such as fibroblasts, our recent data suggest that circulating immune cells respond to mechanical stress and contribute to fibrosis during both cutaneous hypertrophic scar (HTS) and biomedical implant foreign body response (FBR) formation in humans, mice, and pigs (11–14). We have identified that immune cells upregulate Ras-related C3 botulinum toxin substrate 2 (*RAC2)* expression in severe human and murine FBR (13). In a murine FBR model, immune cell-specific knockout and pharmacological inhibition of *RAC2* both significantly reduced FBR and promoted myeloid subpopulations seen in humans. RAC2 is a member of the Rho GTPases, which functions to bind and hydrolyze GTP to regulate a number of critical cell functions and mediate downstream signaling events (15).

**Figure 1:**
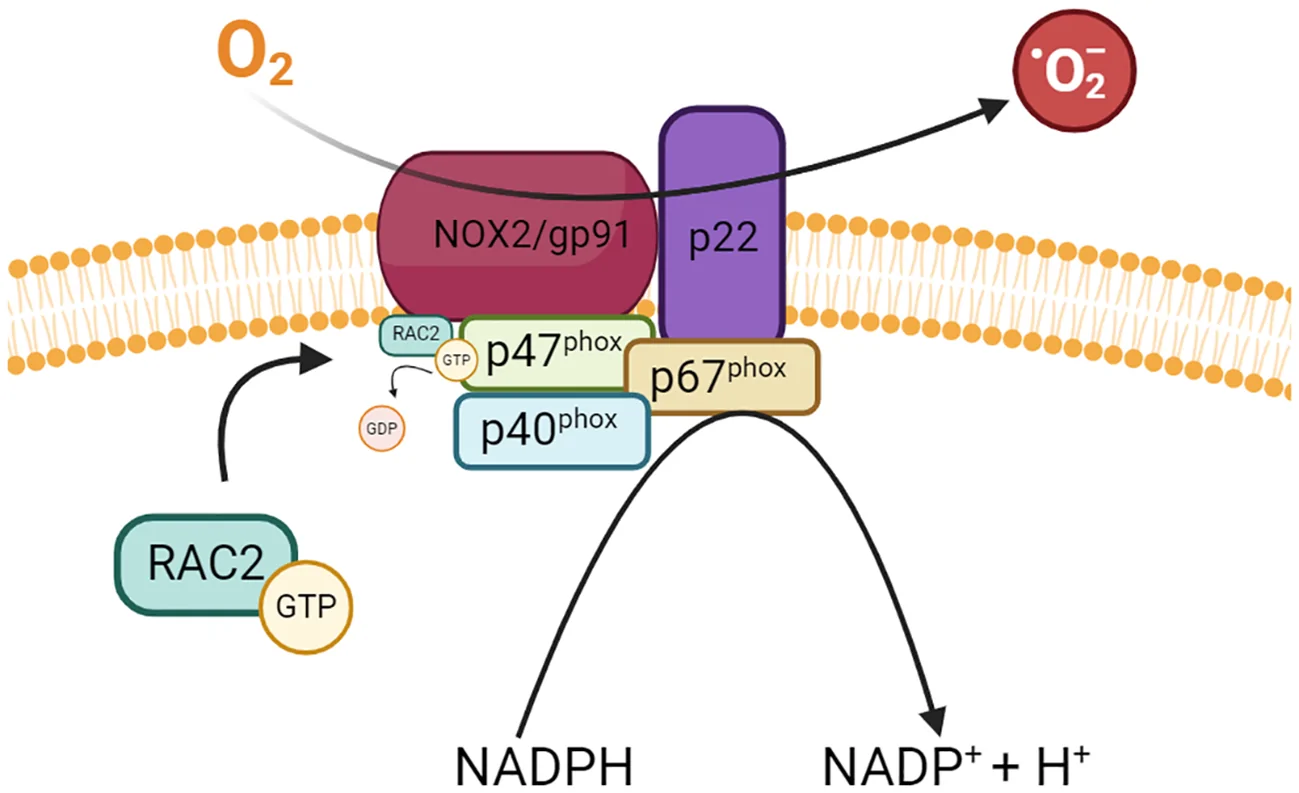
RAC2 plays an essential role in facilitating the coordinated movement of NADPH oxidase components to the membrane surface. Created in https://BioRender.com.

**Figure 2:**
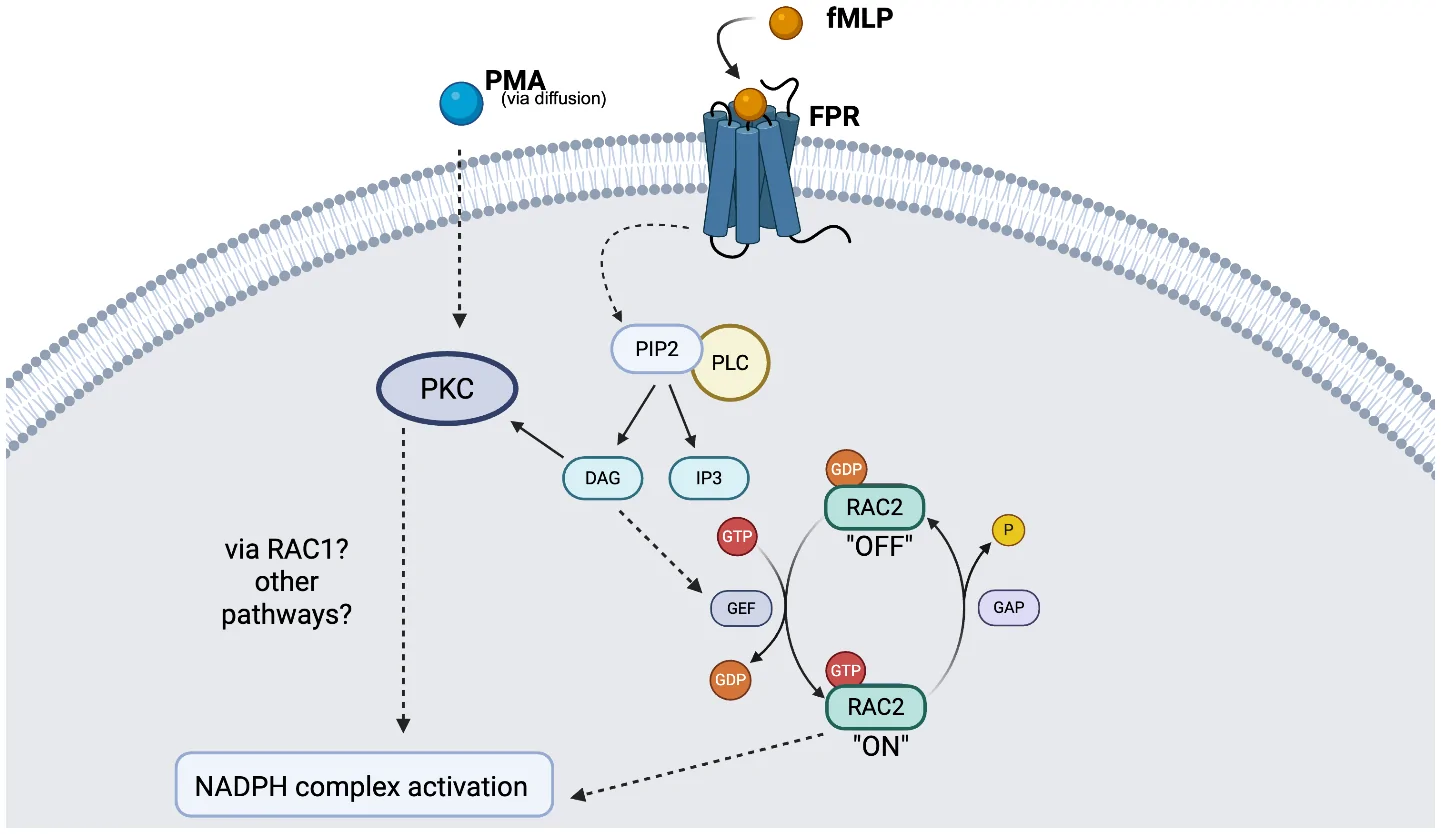
Proposed mechanism for stimulant-specific RAC2 activation of the NADPH oxidase complex in human neutrophils. Created in https://BioRender.com.

To better understand the importance of these highly motile, reactive RAC2+ cells, we performed a comprehensive literature review of RAC2+ innate immune cells across a variety of disease states and organ systems. Here, we summarize the effects of gain-of-function (GOF) and loss-of-function (LOF) *RAC2* mutations on downstream neutrophil phenotypes, such as superoxide production (SO), chemotaxis, and actin polymerization—integral to the wound healing process. We additionally propose a conceptual mechanism by which mutations of *RAC2* may mediate neutrophil biology (**Figures 1**, **2**) and further hypothesize how RAC2 may be implicated in aberrant wound healing and fibrosis. Clinical and laboratory data reviewed in this article are summarized in **Tables 1**, **2**. Although mutations in *RAC2* leading to gain- or loss-of-function have been documented across many case studies and have implications in adaptive and innate immune system dysregulation (16, 17), the literature has yet to propose a link between RAC2, neutrophil biology, and wound healing and fibrosis. Thus, understanding the role of RAC2 in innate immune cells may help elucidate clinical manifestations of the gene mutation, and perhaps identify pathways to target gain- or loss-of-function.

**Table 1:** Summary of clinical case reports and primary literature reporting effects of *RAC2* mutations on neutrophil functions.

RAC2 function	Mutation	Inheritance pattern	Origin	Zygosity	SO fMLP	SO PMA	Chemotaxis	Actin cycling	Ref
Gain	P29R	Autosomal dominant	*De novo*	Het	G	NC	n.d.	L	(18)
	E62K	Autosomal dominant	*De novo*	Het	G	NC	L	G	(19)
					G	NC	L	n.d.	
					G	NC	n.d.	G	
			Inherited		G	n.d.	L	n.d.	(20)
					n.d.	n.d.	L	n.d.	
	Murine E62K	–	–	–	G	G	n.d.	G	(19)
					n.d.	n.d	NC	G	(21)
	N92T	Autosomal dominant	n.d.	Het	n.d.	G	L	n.d.	(22)
	P34H	Autosomal dominant	Inherited familial	Het	G	NC	n.d.	G	(23)
					G	NC	n.d.	G	
					G	NC	n.d.	G	
Loss	D57N	Autosomal dominant	n.d.	Het	NC	G	L	L	(24)
			*De novo*	Het	NC	G	L	L	(25)
			*De novo*	Het	NC	G	L	L	(26)
	Murine KO (*Rac2*^-/-^)	–	–	Homo	n.d.	L	L	L	(27)
					L	L	n.d.	L	(28)
					n.d.	NC	L	L	
Null	W56X	Autosomal recessive	Inherited	Homo	n.d.	n.d.	n.d.	n.d.	(29)
			n.d.	Het	n.d.	n.d.	L	n.d.	(30)

Rows reflect each patient assessed in the referenced study. SO, superoxide production, L, loss, G, gain, NC, no change, n.d., no data, Het, heterozygous, Homo, homozygous.

**Table 2:** Summary of patient clinical features with altered neutrophil phenotypes in reviewed literature.

RAC2 function	Mutation	OMIM classification	Ref	Patient clinical features
Gain	P29R	IMD73B	(18)	11 yo M; RTIs, bronchiectasis
	E62K		(19)	37 yo F; RTIs, pneumonia, bronchiectasis, lymphadenitis, VZV infection, UTIs, cellulitis, pancytopenia, thrombocytopenia
				Newborn F; acute bronchitis, lymphopenia
				14 yo F; chronic rhinitis, recurrent otitis media, recurrent herpes stomatitis, lymphopenia
			(20)	1 yo M; RTIs, reduced PFTs
				adult M; RTIs, pulmonary failure
	N92T		(22)	4 mo F; RTIs, pneumonia, otitis media, COPD, CMV infection, VZV infection
	P34H		(23)	39 yo M; RTIs, bronchiectasis, antiphospholipid syndrome, Non-Hodgkin lymphoma, hypo-IgG
				7 yo F; RTIs, lymphopenia, neutropenia, combined immunodeficiency
				3 yo F; RTIs, lymphopenia, neutropenia, combined immunodeficiency
Loss	D57N	IMD73A	(25)	Newborn M; RTIs, apurulent soft tissue infection, delayed umbilical cord separation, hyperbilirubinemia, thrombocytopenia
			(26)	Newborn M; apurulent peritracheal abscess; CD4 T cell lymphopenia, neutrophilia
Null	W56X	IMD73C	(29)	6 mo F; RTIs, PSGN, membranous glomerulonephritis, Factor XI deficiency, hypogammaglobulinemia, CVID
			(30)	34 yo M; RTIs, bronchiectasis, PSGN, Factor XI deficiency, hypogammaglobulinemia, CVID, PML

OMIM, Online Mendelian Inheritance in Man; IMD, Immunodeficiency; yo, years old; M, male; F, female; RTIs, respiratory tract infection; VZV, Varicella Zoster Virus; UTIs, urinary tract infection; COPD, Chronic Obstructive Pulmonary Disease; CMV, Cytomegalovirus; PSGN, Post-streptococcal glomerulonephritis; CVID, Common Variable Immunodeficiency; PML, Progressive Multifocal Leukoencephalopathy.

## RAC2 activity affects G-protein-mediated superoxide production

Neutrophils are among the first-line defenders of the innate immune system and play a vital role in bacterial and fungal clearance. Neutrophils facilitate host defense by phagocytosis and generation of reactive oxygen species (ROS) in response to chemotactic factors and adhesion to various infection-associated ligands such as Lipopolysaccharide (LPS) (31) and flagellin. The bacterial peptide, *N*-Formyl-methionyl-leucyl-phenylalanine (fMLP), and the chemical, phorbol-12-myristate-13-acetate (PMA), can be used to elicit the generation of ROS via activation and assembly of neutrophil NADPH oxidase components (31). fMLP and PMA are common stimulants used to elicit superoxide production *in vitro (*32).

Upon neutrophil stimulation, 2 cytosolic proteins, p47^phox^ and p67^phox^ translocate to the integral membrane protein, cytochrome b_588_ (NOX2/gp91 and p22 complex). An additional component, Ras-related C3 botulinum toxin substrate (RAC), enzymatically activates the cytochrome b_588_ complex, initiating NADPH oxidase and promoting movement of cytosolic proteins to the cell membrane (33). Together, these events lead to the induction of electron flow through the complex, catalyzing the reduction of O_2_ for generation of superoxide O_2_^-^ and subsequent oxidation of NADPH. Of the RAC-GTPases, RAC2 has been shown to be more active than RAC1, and is the predominant RAC-GTPase expressed in human neutrophils (34). The upregulation of superoxide production is thought be a marker for neutrophil activation and subsequent pathogen clearance (**Figure 1**).

## *RAC2* mutations affect key protein structure and functions

**Figure 3:**
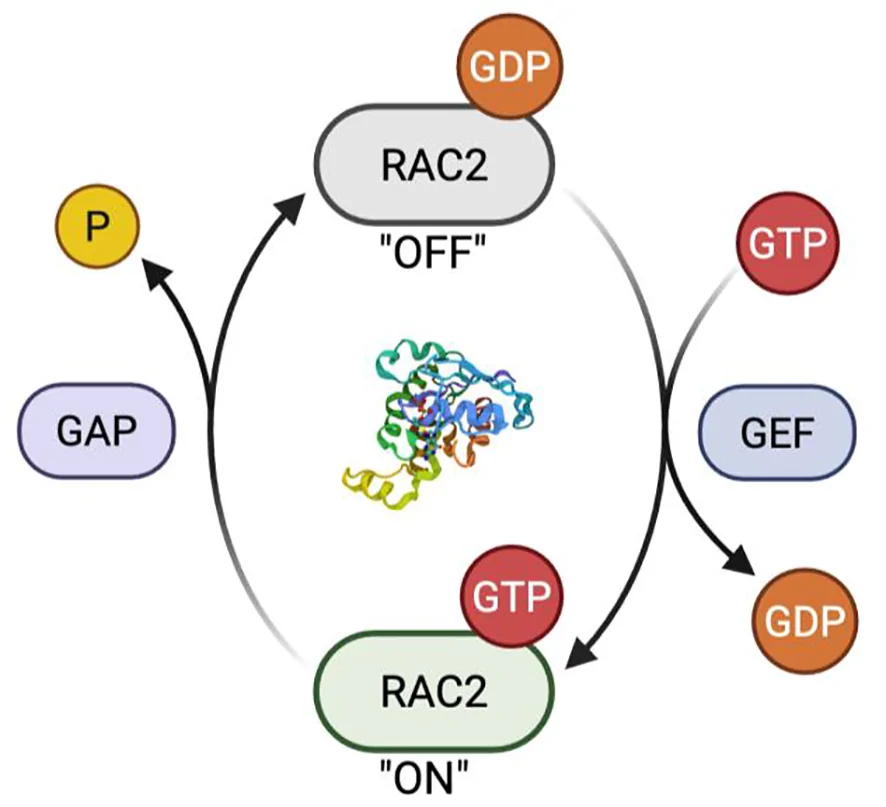
RAC2 is a molecular switch regulated by GTPase-activating proteins (GAPs) and guanine nucleotide exchange factors (GEFs). Created in https://BioRender.com.

RAC2 is considered a molecular “switch”, binding to GTP in its active form and GDP in its inactive form, facilitated by guanine nucleotide exchange factors (GEF), GTPase-activating proteins (GAP), and guanine nucleotide dissociation inhibitor (**Figure 3**). Point mutations associated with clinical disease largely occur within structural regions of the RAC2 catalytic domain, including the guanine nucleotide binding G1 domain/P-loop at residues 10-17, G2 domain/Switch I at residues 25-40, and G3 domain/Switch II at residues 57-75. Indeed, many identified human mutations occur within these regions, conferring loss- or gain-of-function (35).

For instance, the D57N variant (aspartate to asparagine at codon 57), is located within the G3/Switch II domain, conferring domain conformational changes and alterations to the nucleotide-binding pocket (35). The E62K variant, on the other hand, is localized within the G3/Switch II domain, (glutamine to lysine substitution at codon 62) leading to prolonged GTP-bound, active RAC2. Similarly, G12R results in a constitutively active RAC2-GTP (36).

## *RAC2* loss-of-function decreases superoxide generation in humans

The first case report of a patient with a *RAC2* mutation was described in a 5-week-old male who presented in his first week of life with respiratory complications, hyperbilirubinemia, and transient thrombocytopenia (37). At 5 weeks of age, the newborn developed a non-healing nonsuppurative perirectal abscess and omphalitis, suggestive of a neutrophil defect. Interestingly, screening studies for Congenital Granulomatous Disease (CGD), affecting neutrophil and macrophage superoxide (SO) production, and Leukocyte Adhesion Deficiency (LAD), affecting leukocyte chemotaxis, were both negative. Genetic sequencing of the newborn’s monocytes revealed a missense aspartate (D) substitution to asparagine (N) in *RAC2* at exon 3, codon 57. Since the aspartate (D) residue is essential for GTP hydrolysis, this missense mutation caused an attenuation of RAC2 downstream signaling and the protein’s loss of function (LOF). Neither the newborn’s parents, nor sibling carried this mutation, suggesting it was *de novo*.

Neutrophils were collected from peripheral blood and stimulated with fMLP to evaluate superoxide (SO) production. Treated neutrophils revealed reduced SO generation compared to healthy human controls, while PMA stimulation elicited SO production comparable to healthy controls. This finding suggests fMLP and PMA may act through distinct signaling mechanisms in RAC2-dependent and -independent manners, respectively, although this phenomenon appears to be cell type- and species-specific (**Figure 2****).**

The same newborn patient was investigated by Willliams and colleagues, concluding following bone marrow (BM) transplant, fMLP stimulation of the patient’s neutrophils produced elevated SO (24). Successful bone marrow transplantation was demonstrated by variable number tandem repeat (VNTR) analysis, a routine study used to quantify donor engraftment. Prior to transplant, patient neutrophils were also cultured *in vitro* and transduced with murine wildtype (WT) *Rac2*. Interestingly, the introduction of WT *Rac2* did not restore SO production following fMLP treatment, suggesting that D57N is a dominant negative mutation in which downstream functions of RAC2 are unable to be restored with exogenous murine WT *Rac2*. These studies additionally support the hypothesis of RAC2-dependent fMLP stimulation of SO production.

In 2011, another patient was identified with a *RAC2* D57N missense mutation and subsequent LOF (26). A newborn male was diagnosed during newborn screening (NBS) with low T-cell receptor excision circles (TRECs), a quantitative measure of T cell maturity, indicating potential Severe Combined Immunodeficiency (SCID). At 26 days of life, the infant developed a fever and omphalitis. At 56 days, he developed a nonsuppurative peritracheal abscess with few neutrophils. The differential diagnosis included LAD, however, sequencing revealed a sporadic *RAC2* D57N mutation. Assessment of the patient’s neutrophils revealed a similar phenotype to the 5-week-old infant described above (24, 25, 37). In line with previous findings in the infant with a D57N mutation, the patient’s neutrophils also failed to produce SO during fMLP stimulation *in vitro* but demonstrated a normal oxidative burst in response to PMA compared to healthy controls.

Interestingly, a mutation leading to complete absence of RAC2 function has also been observed. *RAC2* W56X is a nonsense mutation at codon 56, leading to a premature stop codon. This mutation was identified in siblings, manifesting as a severe, heterogenous, disease phenotype leading to death in both patients (29, 30). Similar to the infants described with *RAC2* D57N, the clinical presentation of both children, included recurrent pneumonia, nephritis, and a dysfunctional adaptive immune system. Both children were additionally diagnosed with post-streptococcal glomerulonephritis (PSGN), coagulation factor XI (FXI) deficiency, and Combined Variable Immunodeficiency (CVID). Neutrophils from the 6-month-old sister with *RAC2* W56X were not assessed for superoxide production, however, the cells demonstrated decreased and abnormally-sized primary and secondary granules, as well as dense cytoplasmic inclusions and autophagosomes (29). The 2-year-old brother’s neutrophils were assessed for chemotaxis, which was overall reduced (30). These phenotypes suggested severely dysfunctional native neutrophil functions in both patients, which likely contributed to their disease progression and early mortality.

## *Rac2* knockout in murine neutrophils decreases SO production and increases disease susceptibility

*Rac1* and *Rac2* are equally expressed in murine neutrophils, unlike in human neutrophils, which predominantly express *RAC2 (*38). In *Rac2* knockout (KO) mice, the expression of *Rac1* remains entirely unaffected (27). *Rac2* KO models emulate defects of the innate and adaptive immune system similar to those seen in human *RAC2* D57N LOF mutations (27, 28, 39–42). Similar to human infants with nonsuppurative omphalitis and abscesses, *Rac2* KO mice display decreased cellular inflammatory exudate following intraperitoneal thioglycolate injections, potentially due to a lack of neutrophil migration, phagocytosis, and superoxide burst at the site of infection (27). Supporting these findings, *Rac2* KO in a murine colitis model led to worsened disease with an opportunistic bacterial infection, also likely due to dysfunctional neutrophil functions (39). However, in contrast to human cells, neutrophils derived from *Rac2* KO mice demonstrated reduced SO production after treatment with PMA compared to WT controls (27, 28). Heterozygous *Rac2*^+/--^derived neutrophils demonstrated intermediate loss of SO production in response to PMA and fMLP, compared to a more dramatic decrease observed in a complete *Rac2*^-/-^ KO. Because mice express increased *Rac1* compared to humans, these findings confirm that RAC2 plays a predominant role in neutrophil superoxide production and that this phenotype is likely not rescued by RAC1 (28). Taken together, these data suggest a species-specific response to stimulants of SO production in neutrophils (**Figure 2**).

## *RAC2* gain-of-function in neutrophils increases superoxide generation in humans and mice

Gain-of-function (GOF) mutations also alter neutrophil SO production. Activating mutations within the Switch II domain resulting in prolonged GTP-bound RAC2 have been described in a number of patients ranging from newborn to 37 years old (19). All patients demonstrated a history of recurrent opportunistic infections and worsening lung function. In 2019, a 37-year-old female with history of recurrent respiratory tract infections since 2 years of age and an additional history of pancytopenia, lymphadenitis, cellulitis, sepsis, varicella zoster virus (VZV) infection, and frequent urinary tract infections (UTIs) was reported (19). Following whole exome sequencing (WES), the patient was found to have a *de novo* glutamine to lysine missense mutation at codon 62 (E62K), promoting *RAC2* overactivity (GOF). At 42 years old, the patient underwent a successful stem cell transplant. Prior to transplant, the patient’s neutrophils were assessed for SO production following fMLP and PMA activation. Patient-derived neutrophils demonstrated up to a 4-fold increase in SO production when stimulated with fMLP compared to healthy controls. SO production was comparable to heathy neutrophils when stimulated with PMA (19).

Other mutations, such as a missense mutation from proline to arginine at codon 29 (P29R) and proline to histidine at codon 34 (P34H), have been described within the highly conserved Switch I domain of RAC2 and similarly demonstrate constitutive RAC2 activation (23). In 2019, a family with P34H was described, affecting a 39-year-old father and his 7- and 2-year-old daughters. The father first presented at 31 years of age with neutropenia leading to several respiratory opportunistic infections. He later presented with antiphospholipid syndrome, hypo-IgM, and an increased population of senescent T cells (23). His two daughters also presented with multiple sinopulmonary infections, severe lymphopenia, mild intermittent neutropenia, and increased senescent T cells. Both children were diagnosed with combined immunodeficiency. All patient-derived neutrophils with P34H mutation demonstrated an increased oxidative burst with fMLP stimulation, but not with PMA. In 2022, an 11-year-old with a past medical history of recurrent pneumonia since 4 months of age and recurrent episodes of upper and lower respiratory tract infections requiring hospitalizations since 10 years of age was identified via next-generation sequencing (NGS) to have a P29R mutation (18). Patient-derived neutrophils similarly demonstrated increased production of SO in response to fMLP, and comparable SO production to healthy neutrophils when stimulated with PMA.

An additional activating mutation between the *RAC2* Switch II domain and C-terminus was described in 2019, resulting in excessive GTP binding (22). A 4-month-old female first presented with more than 10 episodes of recurrent otitis media. By 5 years old, the child had recurrent pneumonia, Chronic Obstructive Pulmonary Disease (COPD), and spontaneous eardrum perforation. Sequencing revealed *RAC2* asparagine to threonine at codon 92 (N92T), an autosomal recessive *RAC2* GOF mutation. The patient underwent multiple failed stem cell transplants and unfortunately passed away 86 days post-transplant. HEK293 cells transfected with NADPH oxidase components and recombinant *RAC2* N92T demonstrated increased baseline superoxide generation compared to HEK293 cells transfected with WT *RAC2 *(22). Surprisingly, SO generation in *RAC2* N92T transfected cells was increased by PMA stimulation, unlike neutrophils derived from patients with *RAC2* E62K, P29R, or P34H mutations, which did not respond to PMA, suggesting a cell-type specific response to PMA (**Figure 2**) (18, 19, 23). Similar to patient-derived LOF mutation neutrophils, patients with GOF mutations demonstrated aberrant SO production via fMLP-dependent signaling. Interestingly, SO production was also independent of the location of each *RAC2* GOF mutation. *RAC2* E62K, located in the Switch II domain, impairing GDP release/exchange and sustained GTP binding (19), and P29R and P34H, located in the Switch I domain causing constitutive activation of RAC2 via loss of GTP hydrolysis (22), all increased patients’ neutrophil SO production.

Evidence describing constitutively activated RAC2 in a murine model is limited. *Rac2* overactivation has been described in the context of the adaptive immune system, with reduced thymus size and reduced CD4+ and CD8+ T cells in mice (43). More recently, a Cas9 and *Rac2* gRNA model was engineered in murine embryos to derive E62K GOF (19). Neutrophils collected from GOF mice bone marrow demonstrated increased SO production following fMLP stimulation. Fibroblasts cotransfected with NADPH oxidase proteins and recombinant *RAC2* E62K have also been studied to produce a high amount of SO at baseline, which was further increased by PMA stimulation (19).

## Neutrophil RAC2-dependent NADPH oxidase activation is agonist-specific in human neutrophils

Phosphorylation and initiation of cytosolic protein translocation depends on the activity of protein kinase C (PKC), shared by both PMA and fMLP downstream signaling mechanisms (**Figure 2**).

fMLP binds to its receptor, formyl-peptide receptor (FPR), a G-protein coupled receptor (GPCR) expressed on human neutrophils. Activation of FPR produces downstream diacylglycerol (DAG) and inositol 1,4,5-triphosphate (IP3) generation from PIP2 hydrolysis by phospholipase C (PLC). This mobilizes intracellular Ca^2+^ stores to activate PKC and phosphorylate NADPH components (44, 45). Priming neutrophils with fMLP allows time- and dose-dependent activation of SO and other key neutrophil functions (31, 46).

In contrast, PMA diffuses across the neutrophil plasma membrane and activates PKC isoforms, which translocate to the cell membrane and phosphorylate NADPH components (32, 47, 48). Thus, any gain- or loss-of-function of *RAC2* in human neutrophils does not appear to affect downstream signaling mechanisms caused by PMA.

The NADPH oxidase complexes in human neutrophils produce SO or a “respiratory burst” in response to a variety of chemotactic factors (49–51). This oxidative event perpetuates the immune response to pathogens and inflammatory mediators. Therefore, dysfunctional NADPH oxidase results in defective ROS production, leading to recurrent bacterial infections of the skin, respiratory, gastrointestinal, and urinary tracts. Since RAC2 interacts directly with FPR signal transduction, these studies suggest these studies suggest that RAC2-dependent FPR (fMLP GPCR) signaling influence downstream fMLP-mediated SO production to influence downstream fMLP-mediated SO production.

Overall, these findings demonstrate that RAC2 is necessary to promote SO production in a GPCR-dependent fashion in both mice and humans, and that the sensitivity of SO production to various external factors could be dependent on the degree of gene loss or the potential presence of RAC1. In humans, *RAC2* LOF reduces neutrophil superoxide production. Specifically, D57N mutations interrupt the essential GTP binding site of RAC2 and therefore prevent RAC2 from interacting with NADPH oxidase components, while E62K, P34H, P29R, and N92T mutations increase, or over activate GTP binding (27, 52). W56X mutations, on the other hand, completely impair RAC2 function, which is not rescued by WT *RAC2*, suggesting this is a dominant negative mutation. Maintenance of some SO production in LOF mutations may be explained by neutrophil sequestration of guanine nucleotide exchange factors (GEFs) that activate other Rho GTPases, independent of pathways activated by RAC2.

In mice, homozygous *Rac2* knockout demonstrate similar reductions in SO production, although the level of SO generation depends on the level of knockout (e.g. homo- versus heterozygous) (27, 28). LOF *RAC2* in humans and *Rac*2 KO mice displayed similar clinical phenotypes such as lack of suppurative material, or pus, at sites of inflammation or infection, immune dysregulation, and susceptibility to recurrent infections (24–27, 37, 39).

fMLP is currently not an approved therapeutic agent, although several studies have investigated the use of fMLP and activation of FPR to harness the innate immune system in states of infection and in treatment of some diseases, such as hepatitis (53–55). fMLP has also been evaluated as an enhancer of neutrophil ROS production in a mouse model of periprosthetic joint infection caused by *Staphylococcus aureus *(53). This study found that mice injected with fMLP at the site of an infected femoral implant had markedly reduced colony forming units (CFUs) compared to vehicle treatment by day 3 of infection. The investigators also found reduced inflammation in a histological assessment of the femur in fMLP-treated mice. These findings are likely due to neutrophil recruitment and activation following fMLP treatment.

FPR has additionally been described as an essential receptor for neutrophil activation and recognition of *Listeria monocytogenes*, a devastating opportunistic infection reported in immunocompromised and pregnant individuals (55). Interestingly, FPR is also a receptor for a number of other ligands that facilitate antimicrobial and anti-inflammatory functions, including the secretory protein Club Cell Secretory Protein (CC16), found in high abundance in the lung (56, 57). However, physicians utilizing fMLP to activate FPR as a standalone therapy should be wary of its dependence on RAC2 activity, and thus potential ineffectiveness depending on the nature of the variant and the inheritance pattern. Interestingly, *Rac2* KO mice primed with TNF-α partially rescued neutrophil SO burst following treatment with fMLP, compared to mice treated with fMLP alone (27). This finding suggests that in patients with LOF *RAC2* mutations, priming with a pro-inflammatory cytokine or combining therapies with fMLP treatment may be needed to induce SO production similar to healthy individuals.

## RAC2 is essential for neutrophil chemotaxis and actin cytoskeleton reorganization

Neutrophils are among the highly motile innate immune cells essential for pathogen clearance. With increasing concentrations of chemotactic agents, circulating neutrophils roll along the blood vessel wall, extravasate, and migrate towards the site of infection. Filamentous actin (F-actin) is an essential component of the cell cytoskeleton and is particularly important for the mobility of innate and adaptive immune cells. In response to chemoattractants such as fMLP, healthy neutrophils increase density and polymerize F-actin fibers for pseudopod-mediated migration (58, 59). RAC2 is an essential component of the upstream signaling cascade that organizes actin cytoskeleton and development of migrational lamellipodia in neutrophils and other immune cells (60–62). Given the interconnectedness of F-actin and RAC2, we then sought to summarize the effects of *RAC2* LOF and GOF on F-actin formation.

## Loss-of-function *RAC2* and null mutations reduce neutrophil chemotaxis and actin cycling in humans and mice

Neutrophils derived from humans with *RAC2* D57N displayed significantly decreased chemotaxis compared to control neutrophils. Neutrophils from the previously mentioned 1-year-old patient with *RAC2* D57N mutation exhibited aberrant migration in response to various chemoattractants in two studies (24, 25). In response to fMLP and IL-8, few patient-derived neutrophils migrated compared to healthy controls (24). Patient neutrophils similarly demonstrated reduced migration in response to 10% zymosan activated serum (ZAS) (25). Patient neutrophils were evaluated for CD11b expression, which is crucial for adherence and migration in response to chemoattractant (63). Both at rest and in response to stimulation with PMA, fMLP, and platelet-activating factor (PAF), patient neutrophils displayed comparable CD11b expression to healthy control neutrophils (25), not supporting a diagnosis of LAD. Patient *RAC2* D57N-derived neutrophils also demonstrated reduced F-actin formation with an absence of polarization in response to fMLP (25). A similar neutrophil phenotype was observed in the previously described newborn male with *RAC2* D57N presenting with recurrent opportunistic infections, lymphopenia and neutrophilia (26). Patient-derived neutrophils had significantly reduced migration in response to ZAS and further demonstrated disorganized actin without capping or polarization (26).

Null mutations of *RAC2* leading to impacts in neutrophil chemotaxis have also been described (29, 30). A 28-year old man with a history of recurrent sinopulmonary infections, FXI deficiency, CVID, and various autoimmune conditions between the ages of 2 and 16, was identified to have *RAC2* W56X on whole exome sequencing (WES) (29). At age 7, the patient’s neutrophils were isolated and assessed for chemotaxis, which was reduced upon stimulation with chemotactic factor (CF). At 34 years of age, the patient developed a fatal infection with JC virus (JCV), causing progressive multifocal leukoencephalopathy (PML) (30). The patient’s sister similarly presented with a history of recurrent pneumonia, FXI deficiency, post-streptococcal glomerulonephritis (PSGN), CVID, and was confirmed to have *RAC2* W56X on WES (29). The patient’s PSGN ultimately progressed to end-stage renal disease leading to early mortality. While neutrophil chemotaxis was not assessed in this patient, neutrophil phenotype was noted to be severely abnormal, with elongated or collapsed secondary granules with dense cytoplasmic inclusions, hypothesized to be autophagosomes. Considering this abnormal phenotype, it is likely that her neutrophils would have demonstrated abnormal migration and F-actin cycling in response to chemotactic factors, similar to her affected brother (29, 30).

*Rac2* KO models in mice similarly displayed reduced neutrophil chemotaxis in response to various stimuli like fMLP, IL-8, and leukotriene B4 (LTB4) (27, 28). Of the responsive cells, their speed and distance traveled were reduced 4- to 10-fold compared to WT neutrophils (27). Interestingly, expression of FPR in *Rac2* KO neutrophils did not differ from expression of WT neutrophils, suggesting the signaling mechanism upstream of Rac2 was intact. Compared to *Rac2* KO mice, *Rac2*^+/--^derived neutrophils showed only an intermediate dysregulation in migration in response to the same fMLP concentration, suggesting that neutrophil chemotaxis is dependent on the level of *Rac2* gene expression (28). *Rac2* KO mice have also demonstrated a reduction in total neutrophil F-actin concentration prior to stimulation with fMLP, with no change from baseline of relative actin levels over a 30 second period following stimulation (27). *Rac2* KO mice showed reduced total neutrophil count and deficient exudate formation following *in vivo* thioglycolate injections, mirroring findings observed in humans with apurulent wounds, suggesting lack of neutrophil migration, and therefore actin cycling (27).

## *RAC2* GOF results in reduced chemotaxis and increased actin content in human and murine neutrophils

Given *RAC2* LOF decreased actin cycling in neutrophils, we unsurprisingly found that *RAC2* GOF increased actin cycling. Neutrophils from patients with GOF *RAC2* mutations showed elevated F-actin polymerization, although disorganized, compared to LOF *RAC2* patients (18, 19, 23). In two young children with *RAC2* P34H GOF mutations, peripheral blood neutrophils demonstrated increased F-actin content following activation with fMLP (23). Similarly, neutrophils from a patient with *RAC2* P29R GOF mutation demonstrated increased cellular F-actin content with increased cycling between F- and G-actin and impaired polymerization (18).

Although LOF *RAC2* mutations led to decreased neutrophil chemotaxis, strikingly, GOF mutations also decreased chemotaxis. As previously mentioned, GOF *RAC2* leads to overactivity of neutrophil functions like SO production and actin cycling, however, cell motility is negatively impacted. A newborn girl was identified on newborn screening (NBS) to have low T-cell receptor excision circles (TRECs), an indicator of normal thymus development and function (19). Further investigation revealed a *de novo* GOF *RAC2* E62K mutation. The patient experienced acute bronchitis before the age of 1 and underwent hematopoietic stem cell transplant (HSCT) for a successful recovery. Prior to transplant, the patient’s neutrophils were collected and examined for chemotaxis. Surprisingly, the patient’s neutrophils exhibited a reduced response rate and velocity to an fMLP-directed gradient compared to healthy controls, similar to neutrophils with *RAC2* LOF mutations (19). Likewise, a 1-year-old male with recurrent respiratory tract infections and reduced pulmonary function tests (PFTs) was identified to have an activating E62K mutation in addition to his father, with similar clinical features with end stage pulmonary failure. Both the child and his father’s neutrophils demonstrated decreased migratory speed and distance traveled in response to fMLP (20).

Similar to humans, bone marrow-derived neutrophils from a *Rac2* E62K GOF mouse model demonstrated elevated F-actin compared to WT mouse neutrophils (19). In line with this, a recent study utilizing *Rac2 E62K* GOF found F-actin to be significantly elevated, with unchanged chemotaxis in response to CXCL2 compared to WT controls (21). Interestingly, this study noted a biphasic relationship between F-actin and myeloperoxidase (MPO) levels, a highly abundant enzyme found in primary neutrophil granules.

## Proposed mechanism for RAC2-dependent biphasic actin cycling leading to overall reduced cell migration

Prior studies have shown a relationship between extracellular matrix (ECM) stiffness and cell migration, where an optimal ECM stiffness corresponds to optimal cell migration speed. Interestingly, fibroblast migration demonstrates a biphasic correlation with ECM stiffness, wherein both soft and stiff ECM substrates resulted in diminished cell migration in 2D culture (64). *In vivo*, a lack of extracellular ligands would prevent interaction with the ECM and thus, cell migration. Further, *in vivo* mouse models have shown that glioblastoma cells demonstrated reduced migration with increased extracellular ligand expression, associated with decreased cancer progression and better prognosis (65). This mechanical phenomenon has also been mathematically modeled and simulated in 3D culture, but evidence still lacks in the context of humans (66, 67).

**Figure 4:**
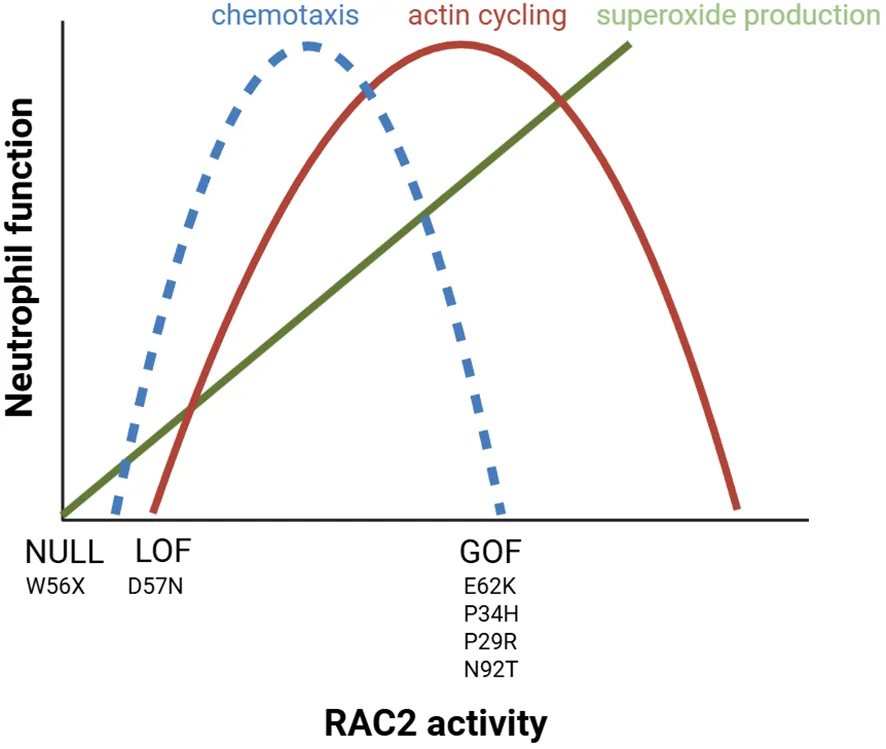
Proposed conceptual mechanism for RAC2-dependent biphasic relationship between neutrophil actin cycling and chemotaxis. Created in https://BioRender.com.

Since RAC2 is upstream of actin cytoskeleton reorganization and cycling, we hypothesize that RAC2 is implicated in a similar biphasic mechanism in human neutrophils (**Figure 4**). In both *RAC2* LOF and GOF mutations, neutrophil chemotaxis is reduced. However, actin cycling is increased in GOF mutations, whereas it is reduced in LOF mutations. Thus, the complex act of cell migration appears to be orchestrated by a delicate balance of mechanical factors, depending on reorganization of the actin cytoskeleton, in response to various mechanical cues and the degree of cell adhesion to the ECM. To our knowledge, we are the first to hypothesize such a mechanism to explain the clinical phenomenon of immune dysregulation and fibrosis due to neutrophil dysregulation.

## The clinical impacts of *RAC2* mutations are heterogenous, warranting a formal clinical diagnosis for severe disease

The *RAC2* gene has been mapped to 22q12.3-q13.2, containing 7 exons, and spanning over 18kb of DNA (68). Mutations leading to GOF or LOF of *RAC2* causing immune dysregulation are recognized by the International Union of Immunological Societies (IUIS) (69). Activated RAC2 defects, for instance, are recognized under “immunodeficiencies affecting cellular and humoral immunity”, specifically, T-B-Severe Combined Immunodeficiency (SCID). Interestingly, congenital B- and T-cell immunodeficiencies can be detected on NBS in the United States, such as SCID and T-cell related lymphocyte deficiencies (70). Although low TRECs are used as a marker to identify newborn patients with immune disorders, congenital neutrophil and phagocyte disorders are not highly considered unless the clinical presentation includes an apurulent wound or recurrent bacterial or viral infections. RAC2 deficiency is recognized under “predominantly antibody deficiencies”, in cases of low IgG, IgA, IgM, B cells, and antibody responses following vaccination. Furthermore, it is classified as a “congenital defect of phagocyte number or function”, if neutrophils are the predominantly affected cell type.

Interestingly, the Online Mendelian Inheritance in Man (OMIM) database recognizes three phenotypic classifications for mutations of the *RAC2* gene located at 22q13.1 (71): Immunodeficiency 73C with defective neutrophil chemotaxis and hypogammaglobulinemia (autosomal recessive), Immunodeficiency 73A with defective neutrophil chemotaxis and leukocytosis (autosomal dominant), and Immunodeficiency 73B with defective neutrophil chemotaxis and lymphopenia (autosomal dominant). Unfortunately, there are currently no clinical trials for Immunodeficiencies 73A, B, or C.

The study of inborn errors of immunity has enabled the classification of disease entities based on clinical phenotypes of immune dysregulation. Identification of clinical sequelae has further allowed for the development of novel therapeutic strategies including hematopoietic stem cell transplantation (HSCT) and gene or pathway editing. Indeed, many patients with *RAC2* mutations identified in infancy have successfully undergone HSCT (72). Large strides toward high throughput, accurate, and cost-effective methods for genetic sequencing have been made since the development of Sanger sequencing in 1977 (73). Almost 40 years later, next-generation DNA sequencing (NGS) technologies like whole exome sequencing (WES) have been utilized extensively for patients with monogenetic diseases (74). WES evaluates protein-coding regions and remains widely used and accepted by clinicians performing genetic screening and counseling (75). Whole genome sequencing (WGS) is becoming increasingly accessible and integrated with artificial intelligence (AI) tools to serve as a comprehensive diagnostic approach (76). Interestingly, recent studies have also identified novel pathways for targeting *RAC2* gain-of-function variants (16). Disorders involving neutrophils with similar clinical phenotypes to those implicated in *RAC2* mutations include Chronic Granulomatous Disease (CGD) and Leukocyte Adhesion Deficiency (LAD). CGD is characterized by defective NADPH oxidase, an X-linked or autosomal recessive disorder affecting neutrophils and macrophages. Mutations in the enzymatic component of NADPH oxidase, gp91phox, account for 70% of all CGD cases (77). LAD I-III characterize the clinical consequences of mutations in neutrophil chemotactic genes such as *CD18*, *CD15*, and *kindlin-3*, respectively (78). These are rare autosomal recessive disorders that present clinically as recurrent bacterial infections without pus, suggesting the lack of neutrophil recruitment to the site of infection. Children with LAD I are typically identified in the first few weeks of life with recurrent infections caused by *Staphylococcus aureus* and *Pseudomonas aeruginosa*. Newborns may even demonstrate delayed separation of the umbilical cord. The clinical manifestations of LAD II are less severe compared to LAD I, although include recurrent skin and upper respiratory infections. Interestingly, cases of newborns and children with *RAC2* mutations present similarly to LAD I-III and CGD, although *RAC2* mutations are not routinely screened for in these clinical scenarios.

Historically, therapeutic options for monogenic disorders have relied on hematopoietic stem cell transplantation (HSCT), providing a curative approach for selected hematologic and immune-related disorders. Recent advances in genome editing and engineering have enabled the development of precision therapies, designed to target and correct disease-causing mutations. CRISPR-Cas technology has emerged as a powerful genome editing tool since its development in 2012 (79), with recent advance investigating non-viral delivery tools. Base editors are an additional therapeutic strategy, avoiding double strand breaks (DSBs) caused by CRISPR-Cas9, with higher editing efficiency and reduced off-target activity (80).

## Limitations of current evidence

Evidence regarding the clinical sequalae associated with *RAC2* mutations in humans is derived from a limited number of case reports. Although these clinical observations are supported by functional and mechanistic studies in mice, the rarity of *RAC2* mutations limit patient recruitment for clinical studies. *RAC2* variants are associated with a spectrum of clinical phenotypes, further complicating genotype-phenotype correlations. Given the severity and disease progression associated with some variants, evidence also lacks long-term outcomes, warranting earlier detection and diagnosis.

## Conclusions

*RAC2* GOF, LOF, and null mutations lead to aberrant neutrophil functions, including superoxide production, chemotaxis, and actin cycling. In both gain- and loss-of-function, neutrophils demonstrated aberrant superoxide production in response to fMLP, which we propose is a RAC2-dependent chemotactic factor, while PMA stimulation led to a normal respiratory burst (**Figure 2**). *RAC2* mutations also facilitated dysregulated actin cytoskeleton remodeling and cycling. In LOF mutations, actin cycling was decreased, leading to reduced migration. Interestingly, GOF mutations similarly led to reduced neutrophil migration and chemotaxis due to overactive actin cycling. We propose that similar migration and chemotaxis outcomes from GOF and LOF mutations are due to a biphasic mechanism of cellular adhesion and migration across the ECM (**Figure 4**). Our findings are the first to hypothesize such a mechanism observed in clinical cases of humans with *RAC2* mutations.
